# Identification of Insecticidal Constituents from the Essential Oil from the Aerial Parts *Stachys riederi* var. *japonica*

**DOI:** 10.3390/molecules23051200

**Published:** 2018-05-17

**Authors:** Meirong Quan, Qi Zhi Liu, Zhi Long Liu

**Affiliations:** Department of Entomology and MOA Key Lab of Pest Monitoring and Green Management, College of Plant Protection, China Agricultural University, Haidian District, Beijing 100193, China; Quanmeirong@cau.edu.cn (M.Q.); lqzwyz@cau.edu.cn (Q.Z.L.)

**Keywords:** *Stachys riederi* var. *japonica*, *Sitophilus zeamais*, *Liposcelis bostrychophila*, fumigant, contact toxicity, essential oil

## Abstract

The essential oil of *Stachys riederi* var. *japonica* (Family: Lamiaceae) was extracted by hydrodistillation and determined by GC and GC-MS. A total of 40 components were identified, representing 96.01% of the total oil composition. The major compounds in the essential oil were acetanisole (15.43%), anisole (9.43%), 1,8-cineole (8.07%), geraniol (7.89%), eugenol (4.54%), caryophyllene oxide (4.47%), caryophyllene (4.21%) and linalool (4.07%). Five active constituents (acetanisole, anisole, 1,8-cineole, eugenol and geraniol) were identified by bioactivity-directed fractionation. The essential oil possessed fumigant toxicity against maize weevils (*Sitophilus zeamais*) and booklice (*Liposcelis bostrychophila*), with LC_50_ values of 15.0 mg/L and 0.7 mg/L, respectively. Eugenol and anisole exhibited stronger fumigant toxicity than the oil against booklice. 1,8-Cineole showed stronger toxicity, and anisole as well as eugenol exhibited the same level of fumigant toxicity as the essential oil against maize weevils. The essential oil also exhibited contact toxicity against *S. zeamais* adults and *L. bostrychophila*, with LC_50_ values of 21.8 µg/adult and 287.0 µg/cm^2^, respectively. The results indicated that the essential oil of *S. riederi* var. *japonica* and its isolates show potential as fumigants, and for their contact toxicity against grain storage insects.

## 1. Introduction

Maize weevil (*Sitophilus zeamais* Motsch.) has a nearly cosmopolitan distribution. Occurring throughout the warmer parts of the world, it is one of the most destructive primary insect pests of stored cereals [1]. It is an internal feeder, and infestations of this weevil not only cause significant losses due to the consumption of grains, they also result in elevated temperature and moisture conditions that lead to an accelerated growth of molds, including toxigenic species [2]. Booklice (*Liposcelis bostrychophila* Badonnel) have also a worldwide distribution, infesting domestic premises, raw material stores, manufacturing factories, and historical documents in museums [3,4]. Currently, psocids are perhaps the most important emerging pests in stored grains and related commodities, due to their small size and resistance to chemicals [5,6]. Control of the stored product insects is currently mainly based on the application of synthetic insecticides/fumigants [7]. However, these insecticides and fumigants are often associated with residues that are dangerous for the consumer and the environment. In addition, repeated use of those fumigants/insecticides for decades has led to resurgence of stored-product insect pests, sometimes resulting in the development of resistance [8,9]. These problems have pushed researchers to find alternative insecticides. In recent years, global research has focused on the possible use of plant secondary metabolites, especially essential oils, for the protection of stored agricultural products. Essential oils and their major constituents, often monoterpenoids, are among the best known substances to have attracted research attention in recent years as potential alternatives to classical fumigants. Investigations in several countries confirm that some plant essential oils not only repel insects, but also possess contact and fumigant toxicity against stored product pests, as well as exhibiting feeding inhibition or harmful effects on the reproductive system of insects [8,9,10,11].

During our mass screening program for new agrochemicals from Chinese medicinal herbs and wild plants, the essential oil of the aerial parts at a flowering stage of *Stachys riederi* var. *japonica* (Miq.) H. Hara (syn. *Stachys japonica* Miq.) were found to possess strong insecticidal activity against maize weevils and booklice. The genus *Stachys* is considered as one of the largest genera of the family Lamiaceae, with nearly worldwide distribution [12]. It comprises about 300 species of annual or perennial herbs and shrubs, and 18 species were found in China [13]. Chinese woundwort (*S. riederi* var. *japonica)* is a perennial, rhizomatous herb distributed only in some areas of China (e.g., Anhui, Fujian, Hebei, Henan, Jiangsu, Jiangxi, Liaoning, Inner Mongol, Shandong, and Zhejiang province), and Russia and Japan. It is distributed in wet areas of ravines and riverbanks, and was used in Chinese traditional medicine for tonsillitis, sore throats, and dysentery [13]. In Korea, this plant has also been used for centuries as traditional medicine to treat homorrhage, coughs, and skin diseases [14]. Chinese woundwort contains caffeic acid, *n*-methoxybaicalein, palustrine, and palustinoside [14,15]. In the previous studies, several phenylethanoid glycosides and oleanane-type triterpene saponins were isolated and identified from this plant [16,17]. However, a literature survey showed that there are no reports on the volatile constituents and insecticidal activity of *S. riederi* var. *Japonica* aerial parts. Thus, we decided to investigate insecticidal activities of the essential oil against two grain storage insects. Also, this paper deals with the isolation and structure determination of bioactive constituents from the essential oil, as well as their insecticidal activities.

## 2. Results and Discussion

### 2.1. GC-MS Analysis

The yellow essential oil yield of *S. riederi* var. *japonica* aerial parts was 0.11% *v*/*w*, and the density of the concentrated essential oil was determined to be 0.83 g/mL. A total of 40 components of the essential oil were identified, representing 96.01% of the total oil composition (Table 1). The principal compounds in *S. riederi* var. *japonica* essential oil were acetanisole (15.43%), anisole (9.43%), 1,8-cineole (8.07%), geraniol (7.89%), eugenol (4.54%), caryophyllene oxide (4.47%), caryophyllene (4.21%), and linalool (4.07%) (Table 1). Monoterpenoids represented 19 of the 40 compounds, corresponding to 41.18% of the whole oil, while 15 of the 40 constituents were sesquiterpenoids (23.86% of the crude essential oil). This is the first documentation of the chemical composition of the essential oil of *S. riederi* var. *japonica* aerial parts. However, the results are quite different from several essential oils derived from Stachys species, which is also found in China. For examples, the major components identified in *S. lanata* essential oil were α-thujone (25.9%), α-humulene (24.9%), β-caryophyllene (12.6%), and viridiflorol (10.5%) [18]. The main compounds in the essential oil of S. palustris from Southern Italy were caryophyllene oxide (7.8%), hexahydrofarnesyl acetone (7.4%), hexadecanoic acid (6.8%), (*Z*,*Z*,*Z*)-9,12,15-octadecatrienoic acid (6.7%), (*Z*)-phytol (6.4%), thymol (5.8%), and *ρ*-methoxyacetophenone (5.1%) [19] while the sample collected from Dolje, Croatia contained 1-octen-3-ol (24.5%), (*E*)-2-hexenal (16.3%), caryophyllene oxide (16.2%), (*E*)-caryophyllene (6.5%), and γ-muurolene (4.8%) [20]. The essential oil of *S. sylvatica* was identified as α-pinene (21.4%), germacrene D (13.6%), β-pinene (12.3%), (*E*)-caryophyllene (9.9%), γ-muurolene (6.4%), and δ-cadinene (4.3%) [21]. However, the most abundant compounds in the oil of *S. sylvatica* from the inflorescences were germacrene D (55.2%), (*E*)-β-farnesene (9.1%), and *n*-tetracosane (6.9%), whilst germacrene D (31.7%), *n*-tetracosane (7.8%) and mint sulphide (6.4%) were the main compounds in the oil from the leaves [21]. The essential oil of *S. sylvatica* from Iran contained β-caryophyllene (19.64%), δ-cadinene (13.41%), spathulenol (12.51%), δ-3-carene (7.38%), and α-copaene (6.38%) [22]. The above findings suggest great variations in chemical composition in the essential oils of the *Stachys* species.

### 2.2. Isolated Bioactive Compounds

**Figure 1:**
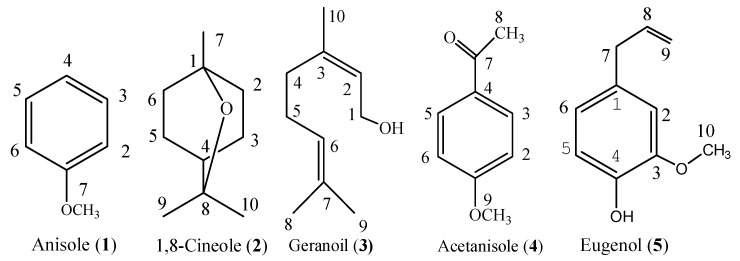
Constituent compounds isolated from the essential oil of *Stachys riederi* var. *japonica* aerial parts.

Purification of the essential oil of *S. riederi* var. *japonica* aerial parts afforded five constituents (Figure 1), the structure of which was elucidated by NMR and MS analysis.

Anisole (**1**, Figure 1), colorless oil. C_7_H_8_O. ^1^H-NMR (500 Hz, CDCl_3_) δ(ppm): 3.76 (3H, 7-CH_3_), 6.88 (1H, 4-H), 6.93 (2H, 2, 6-H), 7.29 (2H, 3, 5-H). ^13^C-NMR (125 Hz, CDCl_3_) δ: 162.0 (C-1), 129.5 (C-3, C-5), 125.3 (C-4), 114.1 (C-2, C-6), 55.1 (C-7). MS *m*/*z* (%): 108 (100), 93 (14), 78 (52), 65 (54), 51 (12), 39 (20). The spectral data matched with the previous reports [23,24].

1,8-Cineole (**2**, eucalyptol, Figure 1), colorless oil. C_10_H_18_O. ^1^H-NMR (500 Hz, CDCl_3_) δ(ppm): 1.05 (3H, 7-CH_3_), 1.24 (6H, 9, 10-CH_3_), 1.41 (1H, 4-H), 1.50 (4H, Ph-H), 1.66 (2H, Ph-H), 2.02 (2H, Ph-H). ^13^C-NMR (125Hz, CDCl_3_) δ: 76.8 (C-8), 72.7 (C-1), 39.6 (C-4), 37.3 (C-2, C-6), 28.9 (C-9, 10), 25.4 (C-7), 24.2 (C-3, 5). MS *m*/*z* (%): 154 (24), 111 (29), 108 (36), 96 (23), 93 (56), 84 (38), 81 (56), 71 (47), 69 (40), 68 (38), 67 (24), 55 (33), 43 (100), 41 (33), 39 (19). The spectral data matched with the previous reports [25,26].

Geraniol (**3**, Figure 1), Colorless oil. C_10_H_18_O. ^1^H-NMR (300 MHz, CDCl_3_) δ (ppm): δ: 5.40 (1H, t, *J* = 6.6, H-2), 5.01 (1H, t, *J* = 6.6, H-6), 4.13 (2H, d, H-1), 2.03 (4H, m, H-4, H-5), 1.67 (6H, s, H-8, 9), 1.60 (3H, s, H-10); ^13^C-NMR (CDCl_3_, 125 MHz) δ (ppm): 139.1 (C-3), 131.6 (C-7), 124.1 (C-2), 123.7 (C-6), 59.2 (C-1), 39.6 (C-4), 26.5 (C-5), 25.7 (C-8), 17.7 (C-9), 16.2 (C-10). EI-MS *m*/*z* (%): 154 ([M]^+^, 4), 136 (8), 123 (11), 93 (30), 69 (100), 68 (19), 67 (10), 41 (68), 39 (11). The spectral data were in agreement with the reported data [25,27].

Acetanisole (**4**, Figure 1). a white solid. C_9_H_10_O_2_. ^1^H-NMR (500 Hz, CDCl_3_) δ(ppm): 2.55 (3H, 8-CH_3_) 3.86 (3H, 7-CH_3_), 6.93 (2H, 2, 6-H), 7.93 (2H, 3, 5-H). ^13^C-NMR (125Hz, CDCl_3_) δ: 196.4 (C-7), 163.5 (C-1), 130.5 (C-3, C-5), 130.3 (C-4), 113.7 (C-2, C-6), 55.3 (C-9), 26.1 (C-8). MS *m*/*z* (%): 150 (30), 135 (100), 107 (20), 92 (24), 77 (38), 64 (18), 43(15). The spectral data matched with the previous reports [23,24].

Eugenol (**5**, Figure 1). a colorless oil, C_10_H_12_O_2_. ^1^H-NMR (CDCl_3_) δ (ppm): 6.83 (1H, d, *J* = 8.8 Hz, H-5), 6.66 (1H, dd, *J* = 8.4, 1.8 Hz, H-6), 6.65 (1H, d, *J* = 1.8 Hz, H-2), 5.94 (1H, m, H-8), 5.73 (1H, br.s, D_2_O exchangeable, -OH), 5.06 (2H, m, H-9), 3.81 (3H, s, -OCH_3_), 3.30 (2H, dt, *J* = 6.6, 1.5 Hz, H-7). ^13^C-NMR (125 MHz, CDCl_3_) δ (ppm): 146.6 (C-3), 144.0 (C-4), 137.9 (C-8), 131.9 (C-1), 115.5 (C-5), 114.5 (C-2), 111.3 (C-9), 55.8 (C-10), 39.9 (C-7). EI-MS *m*/*z* (%): 165 (11), 164 (100), 149 (29), 137 (15), 133 (15), 121 (14), 103 (19), 91 (14), 77 (21), 55 (18). The spectral data matched that given in a previous report [25,28].

### 2.3. Bioactivities

The essential oil of *S. riederi* var. *japonica* exhibited contact toxicity against *S. zeamais* adults and *L. bostrychophila*, with LC_50_ values of 21.8 µg/adult and 287.0 µg/cm^2^, respectively (Table 2 and Table 3). Compared with the famous botanical insecticide, pyrethrum extract, the essential oil was only 5 and 15 times less toxic against maize weevils and booklice, respectively; pyrethrum extract displayed LC_50_ values of 4.3 μg/adult (*S. zeamais*) and 19.0 μg/cm^2^ (*L. bostrychophila*). Among the five isolated constituents, eugenol (LC_50_ = 85.8 μg/cm^2^) showed stronger contact toxicity (no overlap in 95% fiducial limit) than the essential oil against booklice, while acetanisole and geraniol (LC_50_ = 269.2, 270.4 μg/cm^2^, respectively) exhibited the same level of toxicity as the crude oil (Table 2). 1,8-Cineole (LC_50_ = 1076.3 μg/cm^2^) possessed weaker contact toxicity (no overlap in 95% fiducial limit) than the essential oil against booklice. However, anisole did not show any contact toxicity against booklice at current tested concentrations. Similarly, eugenol and 1,8-cineole (LC_50_ = 8.1, 15.8 μg/adult, respectively) showed stronger contact toxicity (no overlap in 95% fiducial limit) than the essential oil against maize weevils, while geraniol (LC_50_ = 18.6 μg/adult) exhibited the same level of toxicity as the crude oil (Table 3). Acetanisole (LC_50_ = 34.0 μg/adult) possessed weaker contact toxicity (no overlap in 95% fiducial limit) than the essential oil against *S. zeamais*, and anisole did not display any contact toxicity against weevils at the tested concentrations (Table 3).

The essential oil of *S. riederi* var. *japonica* aerial parts also possessed fumigant activity against *S. zeamais* and *L. bostrychophila*, with LC_50_ values of 15.0 and 0.2 mg/L air, respectively (Table 2 and Table 3). However, a positive control, methyl bromide (MeBr) was reported to have fumigant activity against *S. zeamais* adults, with an LC_50_ value of 0.7 mg/L air [1]; dichlorvos only has an LC_50_ value of 1.4 μg/L air (Table 3). Thus, the essential oil of *S. riederi* var. *japonica* were only 22 and 163 times less toxic to maize weevil and booklice than the positive controls, respectively. However, compared with the other essential oils with similar bioassays in previous studies, the essential oil of *S. riederi* var. *japonica* exhibited stronger or the same level of fumigant toxicity against maize weevils and booklice, e.g., essential oils of *Ainsliaea fragrans* [29], *Aster ageratoides* [30], *Curcuma wenyujin* [31], *Cyperus rotundus* [31], *Murraya exotica* [32], *Illicium simonsii* [33], *Rhododendron anthopogonoides* [34], and several *Artemisia* species [35,36,37,38]. The above findings suggest that that insecticidal activity, and especially the fumigant activity of the essential oil of *S. riederi* var. *japonica* aerial parts against maize weevils and booklice, is quite promising. As currently used fumigants are synthetic insecticides (e.g., phosphine and MeBr) and are also highly toxic to humans and other non-target organisms, the essential oil of *S. riederi* var. *japonica* aerial parts shows the potential to be used as a natural fumigant/insecticide for the control of the two grain storage insects.

Among five isolates, acetanisole was not found to have fumigant toxicity against the two grain storage insects at the tested concentrations (Table 2 and Table 3). Eugenol and anisole (LC_50_ = 0.1 and 0.5 mg/L, respectively) exhibited stronger fumigant toxicity (no overlap in 95% fiducial limit) than the essential oil against booklice (Table 2). 1,8-Cineole and geraniol (LC_50_ = 1.1 and 1.9 mg/L, respectively) showed weaker fumigant toxicity than the oil against booklice (Table 2). Thus, it seems that fumigant toxicity of the essential oil of *S. riederi* var. *japonica* against booklice may be mainly attributed to eugenol and anisole. Similarly, only 1,8-cineole (LC_50_ = 5.9 mg/L) showed stronger fumigant toxicity (no overlap in 95% fiducial limit) than that of the oil against *S. zeamais* (Table 3). Anisole and eugenol (LC_50_ = 14.8 and 15.9 mg/L, respectively) exhibited the same levels of fumigant toxicity as the essential oil of *S. riederi* var. *japonica* aerial parts against maize weevils (Table 3). The essential oil is normally a mixture of tens to hundreds of individual compounds. Active (insecticidal) ingredients in the essential oil may have different mechanisms of action against insects/mites. Synergistic or antagonistic effects of those active ingredients have been observed in several reports [39,40,41,42,43]. For example, Pavela [39] determined the efficacy of 30 aromatic compounds and their mutual binary combinations for acute toxicity against the larvae of *Spodoptera littoralis*. In total, 435 binary combinations were tested, of which 135 combinations showed a significant synergic effect, while 150 combinations showed a significant antagonistic effect on the mortality of *S. littoralis*. Thus, further studies may need to observe possible synergistic or antagonistic effects of the isolates. On the basis of a literature survey, the three constituents, 1,8-cineole, geraniol, and eugenol, have been shown to possess insecticidal and acaricidal activities, as well as repellency against several insects/mites, including maize weevils (*S. zeamais*) and booklice (*L. bostrychophila*) [21,22,23,44,45,46,47,48,49,50,51,52,53]. However, this is the first report showing that anisole and acetanisole possess insecticidal activities against the two grain storage insects. 

In traditional Chinese and Korean medicine, *S. riederi* var. *japonica* aerial parts are used to treat tonsillitis, sore throats, hemorrhage, coughs, dysentery, and skin diseases [13,14]. It seems that this medicinal herb is quite safe for human consumption, because it has been used as a medicinal herb for hundreds of years. However, no experimental data about the safety of this herb is available so far. Thus, for the practical application of the essential oil as novel fumigant/insecticide, further studies on its safety to humans, and on the development of formulations, are necessary to improve efficacy and stability, and to reduce cost. Moreover, post-application temperature plays a very important role in the compound’s insecticidal efficacy [54]. As the content of the essential oil of *S. riederi* var. *japonica* aerial parts is not high (only 0.111%), for the mass usage of the essential oils as novel botanical insecticide/fumigant, further studies on the effects of increasing of the content of the oil are required.

## 3. Materials and Methods

### 3.1. General

^1^H- and ^13^C-NMR spectra were recorded on Bruker (Billerica, MA, USA) Avance 500, instruments using CDCl_3_ as the solvent with TMS as internal standard. EIMS were determined on a ThermoQuest Trace 2000 mass spectrometer at 70 eV (probe).

### 3.2. Booklice

Booklice, *L. bostrychophila*, were obtained from laboratory cultures, and were maintained in the dark in incubators at 28–30 °C and 70–80% relative humidity, and were reared on a 1:1:1 mixture, by mass, of milk powder, active yeast, and flour. All containers housing insects and the petri dishes used in experiments were made escape proof with a coating of polytetrafluoroethylene (Fluon, Blades Biological Ltd., Edenbridge, Kent TN8 7DX, UK). Adult insects used in all the experiments were about one week old.

### 3.3. Maize Weevils

Maize weevils (*S. zeamais*) were obtained from laboratory cultures, and were maintained in the dark in incubators at 29–30 °C and 70–80% r.h. Maize weevils were reared on whole wheat at 12–13% moisture content in glass jars (diameter 85 mm, height 130 mm). Unsexed adult weevils used in all the experiments were about 2 weeks old.

### 3.4. Plant Material and Essential Oil Extraction

Fresh aerial parts (30 kg) of *S. riederi* var. *japonica* were harvested during the flowering stage in August 2017 from Lishui City (27.54 °N and 119.20 °E, Zhejiang Province, China). The species was identified by Dr Liu, QR (College of Life Sciences, Beijing Normal University), and a voucher specimen (Lamiaceae-*Stachys-riederi-*Lishui-2017-08) was deposited at the Department of Entomology, China Agricultural University (Beijing 100193, China). The aerial parts were cut into pieces and subjected to hydrodistillation using a modified Clevenger-type apparatus for 6 h, before extraction with *n*-hexane. Anhydrous sodium sulphate was used to remove water after extraction. The essential oil was stored in airtight containers in a refrigerator at 4 °C for subsequent experiments.

### 3.5. Gas Chromatography-Mass Spectrometry

The GC analysis of the essential oil of *S. riederi* var. *japonica* aerial parts was accomplished with a Hewlett–Packard 5890 Series II instrument equipped with an HP-5 capillary column (30 m × 0.25 mm, 0.25 μm film thickness), working with the following program: 60° for 10 min, increase of 2 °C/min up to 280 °C; injector and detector temperatures, 270 °C and 300 °C, respectively; carrier gas nitogen 1.0 mL/min; detector dual FID, split ratio 1:30; injection of 0.5 μL. GC-MS analysis of the essential oil of *S. riederi* var. *japonica* aerial parts was performed with a Agilent 6890N (Agilent Technologies, Santa Clara, CA, USA) gas chromatograph connected to Agilent 5973N mass selective detector at 70 eV ionization energy, equipped with an HP-5MS capillary column (5% diphenyl and 95% dimethylpolysyloxane, 30 m × 0.25 mm × 0.25 μm). Helium was used as a carrier gas at a flow rate of 1.0 mL/min. Oven temperature was programmed as from 60 °C to 180 °C at 10 °C/min, remaining at 180 °C for 1 min, and then ramped up by 20 °C/min to 280 °C, and held there for 15 min. The injector temperature was held at 270 °C. The samples (1 μL, 1% acetone solution) were instilled, with a split ratio of 1:10. Spectra were scanned over the range 20 to 550 *m*/*z* at 2 scans s^−1^. Identification of most constituents were based on comparison of their retention indices with those reported in the literature, or with those of authentic compounds available in our laboratories. Retention indices were determined using retention times of *n*-alkanes (C_8_–C_24_) under the same chromatographic conditions. Further identification was made by comparison of their mass spectra with those of samples stored in NIST 05 (Standard Reference Data, Gaithersburg, MD, USA) and Wiley 275 libraries (Wiley, New York, NY, USA), or with mass spectra from the literature [55]. Component relative percentages were calculated based on GC peak areas without using correction factors.

### 3.6. Bioactivity-Directed Isolation

The crude essential oil of *S. riederi* var. *japonica* (30 mL) was purified on a silica gel [Merck 9385 (Merck &Co., Inc. Darmstadt, Germany), 1000 g] column (95 mm i.d., 900 mm length) by gradient elution with a mixture of solvents (*n*-hexane:ethyl acetate = 100:0–0:100, *v*/*v*). Fractions (500 mL each) were collected and concentrated at 40 °C, and similar fractions according to thin layer chromatography (TLC) profiles were combined to yield 14 fractions. Based on contact bioassay, fractions (**3**, **5**, **7**, **9** and **12**) were further separated by preparative silica gel column chromatography (PTLC) to obtain five pure compounds; anisole (0.35 g), 1,8-cineole (0.42 g), geraniol (0.27 g), eugenol (0.21 g), and acetanisole (0.19 g).

### 3.7. Contact Toxicity

The contact toxicity of the essential oil and its isolated constituents against maize weevils (*S. zeamais*) adults was measured using a topical application method, as described by Liu and Ho [1]. Range-finding studies were run to determine the appropriate testing concentrations. A serial dilution of the essential oil/compounds (six concentrations, 2.0–30.0%, *v*/*w*) was prepared in *n*-hexane. Aliquots of 0.5 μL of the dilutions were applied topically to the dorsal thorax of the weevils, using a Burkard Arnold microapplicator (Burkard Manufacturing Co. Ltd., Rickmansworth, London, UK). Pyrethrum extract was used as a positive control. Controls were determined using 0.5 µL of *n*-hexane per weevil. Ten insects were used for each concentration and control, and the experiment was replicated six times. Both treated and control insects were then transferred to glass vials (10 insects/vial) with culture media and kept in incubators at 29–30 °C and 70–80% relative humidity. Mortality recordings were taken after 24 h of exposure. Pyrethrum extract (25% pyrethrin I and pyrethrin II) was purchased from Fluka Chemie, Buchs, Switzerland.

The contact toxicity of the essential oil/constituents against booklice (*L. bostrychophila*) was measured by an impregnated filter paper method, as described by Zhao et al. [47]. Range-finding studies were run to determine the appropriate testing concentrations. The essential oil and isolated constituents were diluted in acetone. The filter paper with a diameter of 3.5 cm (Whatman) was treated with 150 μL of the solution. After treatment with solid glue (Glue Stick, Jong Ie Nara Co., Ltd., Hong Kong, China), the filter paper was placed in a Petri dish (3.5 cm in diameter) and 10 booklice were onto it using a hair brush. A plastic cover with holes was placed over it, and all the Petri dishes were kept in incubators at 27–29 °C and 70–80% relative humidity for 24 h. Acetone was used as the control, and pyrethrum extract was used as the positive control. Five concentrations and five replicates of each concentration were used in all treatments and controls. Mortality recordings were taken after 24 h of exposure.

### 3.8. Fumigant Toxicity

The fumigant toxicity of the essential oil and isolates against *S. zeamais* adults was recorded, as described by Liu and Ho [1]. Range-finding studies were run to determine appropriate testing concentrations. A serial dilution of the essential oil/pure compounds (2.0–40.0%, six concentrations) was prepared in *n*-hexane. A Whatman filter paper (diameter 2.0 cm) was placed on the underside of the screw cap of a glass vial (diameter 2.5 cm, height 5.5 cm, volume 24 mL). Ten microliters of an appropriate concentration of the essential oil/compounds was added to the filter paper. The solvent was allowed to evaporate for 10 s before the cap was placed tightly on the glass vial (with 10 insects) to form a sealed chamber. Fluon (ICI America Inc., Wilmington, DE, USA) was used inside each glass vial to prevent weevils from coming into contact with the treated filter paper. Preliminary experiments demonstrated that 10 s were sufficient for the evaporation of solvents. *n*-Hexane was used as a control. Six replicates were used in all treatments and controls; they were incubated at 29–30 °C and 70–80% r.h. for 24 h, and the mortality was recorded.

The fumigant toxicity of the essential oil/constituents against booklice (*L. bostrychophila*) was determined, as described by Zhao et al. [52]. Range-finding studies were run to determine the appropriate testing concentrations of the pure compounds and *S. riederi* var. *japonica* essential oil. A filter paper strip (3.5 cm × 1.5 cm) was treated with 10 μL of an appropriate concentration of test essential oil/compound in acetone. The impregnated filter paper was then placed in the bottom cover of glass bottle of 250 mL. The insects, 10 adults in a small glass bottle (8 mL), were exposed for 24 h and each concentration with five replicates. All the treatments were replicated five times. Acetone was used as controls and dichlorvos was used as a positive control. The observed mortality data were corrected for control mortality using Abbott’s formula. A positive control, dichlorvos (99.9%) was purchased from Aladdin-reagent Company (Shanghai, China).

### 3.9. Data Analysis

Mortality of insects was observed and the observed data were corrected for control mortality, using Abbott’s formula. The results from all replicates were subjected to probit analysis, using the PriProbit Program V1.6.3 to determine LC_50_ values [56]. Significant differences in LC_50_ and LC_90_ were based on nonoverlap of the 95% confidence intervals (FL).

## 4. Conclusions

The study indicates that the essential oil of *S. riederi* var. *japonica* aerial parts and its constituent compounds have potential for development into natural insecticides/fumigants for the control of insects in stored grains

## Figures and Tables

**Table 1:** Volatile compounds identified in the essential oil derived from *Stachys riederi* var. *japonica* aerial parts.

	RI *	Compound	Composition, %
	Monoterpenoids		41.18
**1**	931	α-Pinene	1.23
**2**	974	β-Pinene	1.43
**3**	991	β-Myrcene	1.02
**4**	1016	δ-3-Carene	0.68
**5**	1025	*ρ*-Cymol	0.32
**6**	1029	Limonene	1.56
**7**	1032	1,8-Cineole	8.70
**8**	1037	(*Z*)-β-Ocimene	0.77
**9**	1067	*cis*-Linalool oxide	1.08
**10**	1091	Terpinolen	0.49
**11**	1094	Linalool	4.07
**12**	1146	Camphor	0.65
**13**	1174	Borneol	3.25
**14**	1179	4-Terpineol	1.43
**15**	1182	*ρ*-Cymen-8-ol	0.38
**16**	1189	α-Terpineol	3.85
**17**	1236	Pulegone	0.21
**18**	1252	Geraniol	7.89
**19**	1287	Bornyl acetate	2.17
	Sesquiterpenoids		23.86
**20**	1375	α-Copaene	0.23
**21**	1387	β-Cubebene	1.33
**22**	1420	Caryophyllene	4.21
**23**	1430	β-Copaene	0.89
**24**	1485	Germacrene D	1.82
**25**	1492	δ-Selinene	0.83
**26**	1454	α-Caryophyllene	1.33
**27**	1523	δ-Cadinene	0.67
**28**	1538	Dihydroactinolide	1.61
**29**	1578	Spathulenol	2.55
**30**	1583	Caryophyllene oxide	4.47
**31**	1598	α-Cedrol	1.91
**32**	1631	γ-Eudesmol	1.18
**33**	1642	τ-Muurolol	0.45
**34**	1648	β-Eudesmol	0.38
	Phenylpropanoids		4.88
**35**	1195	Estragole	0.34
**36**	1356	Eugenol	4.54
	Others		26.09
**37**	923	Anisole	9.23
**38**	993	3-Octanol	1.11
**39**	1065	Acetophenone	0.32
**40**	1348	Acetanisole	15.43
	Total identified		96.01

* RI, retention index as determined on a HP-5MS column using the homologous series of *n*-hydrocarbons.

**Table 2:** Contact and fumigant toxicity of the essential oil of *Stachys riederi* var. *japonica* aerial parts against the adults of *Liposcelis bostrychophila.*

Toxicity	Treatment	LC_50_ (95% FL)	LC_90_ (95% FL)	Slope ± SD	Chi-Square Value
Contact toxicity (μg/cm^2^)	Essential oil	287.0 (269.8–303.6)	538.9 (507.2–571.3)	5.56 ± 0.64	6.47 *
	Acetanisole	269.2 (253.7–284.5)	514.8 (382.2–448.4)	6.82 ± 0.72	11.32 *
	Anisole	>1500	-	-	-
	1,8-Cineole	1076.3 (987.0–1154.3)	1769.5 (1634.6–1912.7)	9.20 ± 0.99	20.74
	Eugenol	85.8 (78.3–92.5)	155.1 (141.6–168.4)	7.51 ± 0.72	9.08 *
	Geraniol	270.4 (249.1–296.5)	611.9 (564.7–662.0)	9.61 ± 0.97	16.24 *
	Pyrethrum extract	19.0 (17.6–20.1)	48.2 (45.5–52.4)	7.64 ± 1.05	9.45^*^
Fumigant (mg/L)	Essential oil	0.7 (0.6–0.8)	2.0 (1.8–2.0)	2.93 ± 0.31	15.46 *
	Acetanisole	>80	-	-	-
	Anisole	0.5 (0.4–0.5)	0.9 (0.8–0.9)	8.39 ± 0.81	12.99 *
	1,8-Cineole	1.1 (1.0–1.2)	2.0 (1.8–2.1)	4.80 ± 0.50	12.70 *
	Eugenol	0.1 (0.1–0.2)	0.3 (0.2–0.3)	6.43 ± 0.62	9.47 *
	Geraniol	1.9 (1.8–2.1)	3.8 (3.5–4.1)	7.35 ± 0.75	23.24 *
	Dichlorvos	1.4 × 10^−3^ (1.3–1.5) × 10^−3^	4.6 × 10^−3^ (4.2–4.9) × 10^−3^	6.87 ± 0.77	4.27 *

* Values were significant at the *p* < 0.05 level.

**Table 3:** Contact and fumigant toxicity of the essential oil of *Stachys riederi* var. *japonica* aerial parts against the adults of *Sitophilus zeamais*.

Toxicity	Treatment	LC_50_ (95% FL)	LC_90_ (95% FL)	Slope ± SD	Chi-Square Value
Contact toxicity (μg/adult)	Essential oil	21.8 (19.5–23.2)	43.5 (39.4–47.9)	9.19 ± 0.84	18.48 *
	Acetanisole	34.0 (30.0–36.8)	42.4 (38.5–46.3)	7.33 ± 0.71	9.84 *
	Anisole	>150	-	-	-
	1,8-Cineole	15.8 (15.3–16.3)	27.8 (25.3–30.1)	4.06 ± 0.37	12.97^*^
	Eugenol	8.1 (7.3–8.8)	17.5 (16.1–19.1)	5.75 ± 0.47	11.34 *
	Geraniol	18.6 (16.8–20.0)	31.5 (29.3–34.6)	5.53 ± 0.55	15.40 *
	Pyrethrum extract	4.3 (3.9–4.7)	8.2 (45.5–52.4)	4.64 ± 0.35	9.45 *
Fumigant (mg/L)	Essential oil	15.0 (13.8–16.4)	35.5 (32.6–38.3)	7.72 ± 0.75	17.64 *
	Acetanisole	>80	-	-	-
	Anisole	14.8 (13.7–16.4)	34.04 (31.34–37.21)	6.17 ± 0.57	13.04 *
	1,8-Cineole	5.9 (5.5–6.4)	12.8 (11.9–14.2)	7.26 ± 0.70	13.46 *
	Eugenol	15.9 (14.6–17.2)	37.5 (34.0–40.3)	6.45 ± 0.52	12.29 *
	Geraniol	19.2 (17.6–20.8)	42.2 (38.8–45.0)	5.15 ± 0.42	14.65 *
	MeBr **	0.7	-	-	-

* Values were significant at the *p* < 0.05 level. ** Data from Liu and Ho [1].
